# Distinct microbial and functional alterations across skin sites and disease severity in pediatric atopic dermatitis: a prospective study

**DOI:** 10.3389/fmed.2026.1805596

**Published:** 2026-04-29

**Authors:** Zirui Feng, Heng Quan, Mao Li, Dan He, Yujie Han, Chenghui Zou, Wen Zhang, Jing Chang, Mao Lu

**Affiliations:** 1 School of Clinical Medicine, Chengdu Medical College, Chengdu, China; 2 The First Affiliated Hospital of Chengdu Medical College, Chengdu, China; 3 Jiulongpo People’s Hospital, Chongqing, China; 4 Sichuan Milan Bravou Medical Beauty Hospital, Chengdu, China

**Keywords:** atopic dermatitis, functional analysis, high-throughput nucleotide sequencing, metagenomic analysis, skin microbiome

## Abstract

**Background:**

Atopic dermatitis (AD) is a chronic inflammatory skin condition frequently associated with microbial dysbiosis.

**Objective:**

This study examined the diversity, composition, and functional profiles of the skin microbiome in children with varying degrees of AD in different skin regions.

**Methods:**

Skin samples were collected from 12 AD patients and 12 healthy controls. Genomic DNA underwent shotgun metagenomic sequencing to analyze alpha and beta diversity, taxonomic composition, and functional profiles, including the Kyoto Encyclopedia of Genes and Genomes (KEGG), Gene Ontology (GO), virulence factors and pathogen-host interactions (PHI).

**Results:**

Significant differences were observed in Shannon’s diversity index and Chao1 diversity index between severity groups (*p* = 0.007 and 0.004). Cluster analysis revealed partial clustering by severity, with significant differences between mild and moderate groups (*p* = 0.042) and between moderate and severe groups (*p* = 0.036). *Staphylococcus* and *Streptococcus* dominated the abundance profile in AD samples. Functional analysis revealed alterations in epidermal microbial activity during AD onset and across different severity levels.

**Conclusion:**

Pediatric AD involves site- and severity-specific microbial shifts. This functional dysregulation and enrichment of virulence factors may push barrier dysfunction and inflammation, suggesting that the microbiome is a critical target for future therapies.

## Introduction

Atopic dermatitis (AD) is a common chronic dermatologic condition characterized by recurrent eruptions, dry skin, and pruritus (1). As of 2022, approximately 200 million individuals worldwide are affected by AD (2). AD manifests heterogeneously, presenting with erythema, papules, nodules or lichenification, often accompanied by varying degrees of itch, all of which substantially impair quality of life (3, 4). Moreover, AD is frequently comorbid with other atopic and infectious conditions such as rhinitis, asthma, and cutaneous microbial infections (5). Poor treatment adherence and the absence of a definitive cure render AD management particularly challenging (6, 7).

The pathogenesis of AD is multifactorial, involving genetic mutations, environmental exposures, and alterations in the cutaneous microbiome (8). Genetically, mutations in genes such as filaggrin (FLG) are associated with the development of AD, as they disrupt keratinocyte organization and compromise the skin barrier (9, 10). Environmentally, factors such as tobacco smoke exposure, alcohol consumption, and dietary deficiencies have been identified as risk contributors (11). Moreover, dysbiosis of the skin microbiota is recognized as a key driver in AD pathogenesis (12). Specific microbes such as *Staphylococcus aureus* (*S. aureus*) can drive the onset and progression of AD by producing bacterial toxins, expressing adhesion factors, and inducing the accumulation of inflammatory cells (13, 14).

Although multiple etiologic mechanisms have been implicated, objective evidence linking the microbial community and its function to AD in pediatric patients remains scarce. Metagenomic sequencing offers a promising approach to fill this gap. Metagenomics leverages high-throughput sequencing to profile the taxonomic composition and relative abundance of microorganisms within a given sample (15). It has been revealed that *α*-diversity is reduced in AD lesions compared to healthy controls, with partial restoration post-treatment and dominance by *Staphylococcus*, *Pseudomonas*, and *Streptococcus* species by metagenomic methods (16, 17). Furthermore, *S. aureus* is significantly overrepresented in lesional versus nonlesional skin (17). However, existing metagenomic studies of AD have primarily focused on comparisons between patients with AD and healthy controls, with limited investigation of differences across disease severity levels and skin sampling sites. This study aimed to investigate differences in microbial diversity and species richness among pediatric patients with AD, stratified by site of onset and disease severity, and to further examine alterations in microbial functional profiles across different sites and levels of disease severity through functional analysis.

## Materials and methods

### Study population

This study was conducted in accordance with the Declaration of Helsinki and was approved by the Ethics Committee of the First Affiliated Hospital of Chengdu Medical College (Approval No. 2022CYFYIRB-BA-Jul04). Participants were recruited between January and June 2023 at the First Affiliated Hospital of Chengdu Medical College. The inclusion criteria for patients with AD were as follows: (1) aged between 2 and 11 years, (2) written informed consent was voluntarily obtained from a parent or legal guardian, and (3) newly diagnosed with AD based on Williams’ criteria (18). Exclusion criteria for AD patients were: (1) presence of serious systemic comorbidities, (2) history of medication or conditions related to skin diseases, (3) use of antibiotic topical products or medications for at least 1 month, and (4) absence of AD lesions at any of the three target sites, including the cheek, antecubital fossa, and popliteal fossa. AD participants underwent blinded Scoring of Atopic Dermatitis (SCORAD) assessment and were stratified into mild, moderate, or severe subgroups based on their scores (19). Disease severity was categorized based on the SCORAD score as follows: <25, mild; 25–50, moderate; and >50, severe. Healthy controls were recruited through open invitations and were confirmed to have no personal or family history of dermatological diseases.

### Skin sample collection

All participants underwent sampling on the day following their inclusion in the study group. Participants were instructed to avoid bathing and using any antimicrobial personal care products within 24 h prior to sampling. No cleansing or topical applications were performed at the sampling sites before specimen collection. Sterile swabs pre-moistened with phosphate-buffered saline were rubbed back and forth across bilateral cheek, antecubital, and popliteal regions, and subsequently labeled by anatomical site. Samples were immediately flash-frozen at −80 °C and shipped in light-protected containers packed with dry ice.

### Microbiome sequencing and data processing

Following sample storage and shipment, microbial genomic DNA was extracted from the swabs using the ZymoBIOMICS™ DNA Microprep Kit (Zymo Research, USA). Subsequently, DNA concentration and purity were assessed using the Qubit™ dsDNA HS Assay Kit on a Qubit 4.0 fluorometer (both from Thermo Fisher Scientific, USA). Shotgun sequencing libraries were then prepared with the VAHTS® Universal Plus DNA Library Prep Kit (Nanjing Novizan Biotech Co., Ltd., China), followed by evaluation of fragment size distribution and integrity on an Agilent 2,100 Bioanalyzer using the High Sensitivity DNA Kit (Agilent Technologies, USA). Next, paired-end sequencing (2 × 150 bp) was conducted on the DNBSEQ-T7 platform (Shenzhen Huada Zhizao Science & Technology Co., Ltd., China) at Chengdu Life Baseline Technology Co., Ltd. Raw sequencing reads were quality-filtered with BBDuk (version 39.01), retaining only reads exceeding 50 bp in length. Host-derived sequences were depleted by alignment to the human hg19 reference genome using Bowtie2 (version 2.3.5.1), and only non-host reads were retained for downstream taxonomic and functional annotation.

Following sequence depletion, the filtered reads from each sample were *de novo* assembled into contigs using MEGAHIT (version 1.2.9). Open reading frames (ORFs) were predicted within these contigs with Prodigal (version 2.6.3) and the predicted genes from all samples were clustered using CD-HIT (version 4.8.1) to construct a non-redundant gene catalog. The quality-filtered reads were mapped back to this gene catalog with Bowtie2 (version 2.3.5.1) to calculate reads per kilobase per million mapped reads (RPKM) values for gene abundance estimation.

### Taxonomic and functional gene annotation

Taxonomic classification was performed on the quality-filtered reads using MetaPhlAn (version 4.1.1) to profile microbial community composition and estimate relative abundances. Taxonomic results were primarily summarized at the genus level. Functional annotation of the predicted protein sequences was conducted using DIAMOND blastp alignments against the virulence factor database (VFDB; release 20,230,224) to detect virulence factors (20). Additionally, gene ontology (GO) terms were assigned, while metabolic pathways were annotated by mapping sequences to the Kyoto Encyclopedia of Genes and Genomes (KEGG) database (21). Pathogen–host interactions (PHI) analysis was performed by comparing the gene set against the pathogen–host interactions database (PHI-base) using basic local alignment search tool for proteins (BLASTP).

### Statistical analysis

All statistical analyses were performed using the vegan package (version 2.5–7) in R software (version 4.3.2), with a significance threshold set at *p* < 0.05 (22). Data normality was assessed via the Shapiro–Wilk test prior to differential analyses. Alpha diversity was quantified using three indices: Shannon index, Chao1 estimate, and Simpson index. Beta diversity was evaluated based on Bray–Curtis dissimilarity matrices and visualized through principal coordinate analysis (PCoA), with group differences tested using permutational multivariate analysis of variance (PERMANOVA). For differential taxonomic abundance, linear discriminant analysis effect size (LEfSe) was applied with thresholds of LDA score ≥ 2.0 (23). Relative microbial abundances were compared between two groups using Student’s t-test if data were normally distributed or the Mann–Whitney U test otherwise; for comparisons across more than two groups, one-way ANOVA or the Kruskal–Wallis test was employed, followed by Tukey’s or Dunn’s *post hoc* tests with Benjamini–Hochberg correction for multiple comparisons, respectively. The analysis was calculated at the individual-sample level. Similarly, alpha diversity indices were compared using the same parametric or non-parametric approaches based on normality assumptions.

## Results

### Demographic characteristics

This study enrolled a total of 24 participants, including 12 patients with AD and 12 healthy controls, as shown in Table 1. The AD patient group consisted of 7 males and 5 females, with ages averaging 5.33 ± 3.09 years, ranging from 2 to 11 years. The healthy control group included 6 males and 6 females, with ages averaging 5.75 ± 1.96 years, ranging from 3 to 9 years. There was no significant difference in age between the two groups (t_22_ = −0.39, *p* = 0.70). Similarly, there was no significant difference in gender distribution between the two groups (*χ*^2^ = 0.17, *p* = 0.68). The mean SCORAD score for the 12 AD patients was 38.43 ± 15.49 points, ranging from 17.70 to 61.80 points. Samples were successfully collected from all three skin sites in all pediatric patients with AD.

**Table 1:** Baseline characteristics of the patients and healthy controls enrolled in the study.

Variable	Atopic dermatitis group	Healthy control group	*p*
Age (mean ± SD)	5.33 ± 3.09	5.75 ± 1.96	0.697
Gender			0.682
Male	7	6	
Female	5	6	
Disease severity			—
Mild	4	—	—
Moderate	4	—	—
Severe	4	—	—
SCORAD score (mean ± SD)	38.43 ± 15.49	—	—

*p* < 0.05 was considered significant.

### Diversity analysis

**Figure 1:**
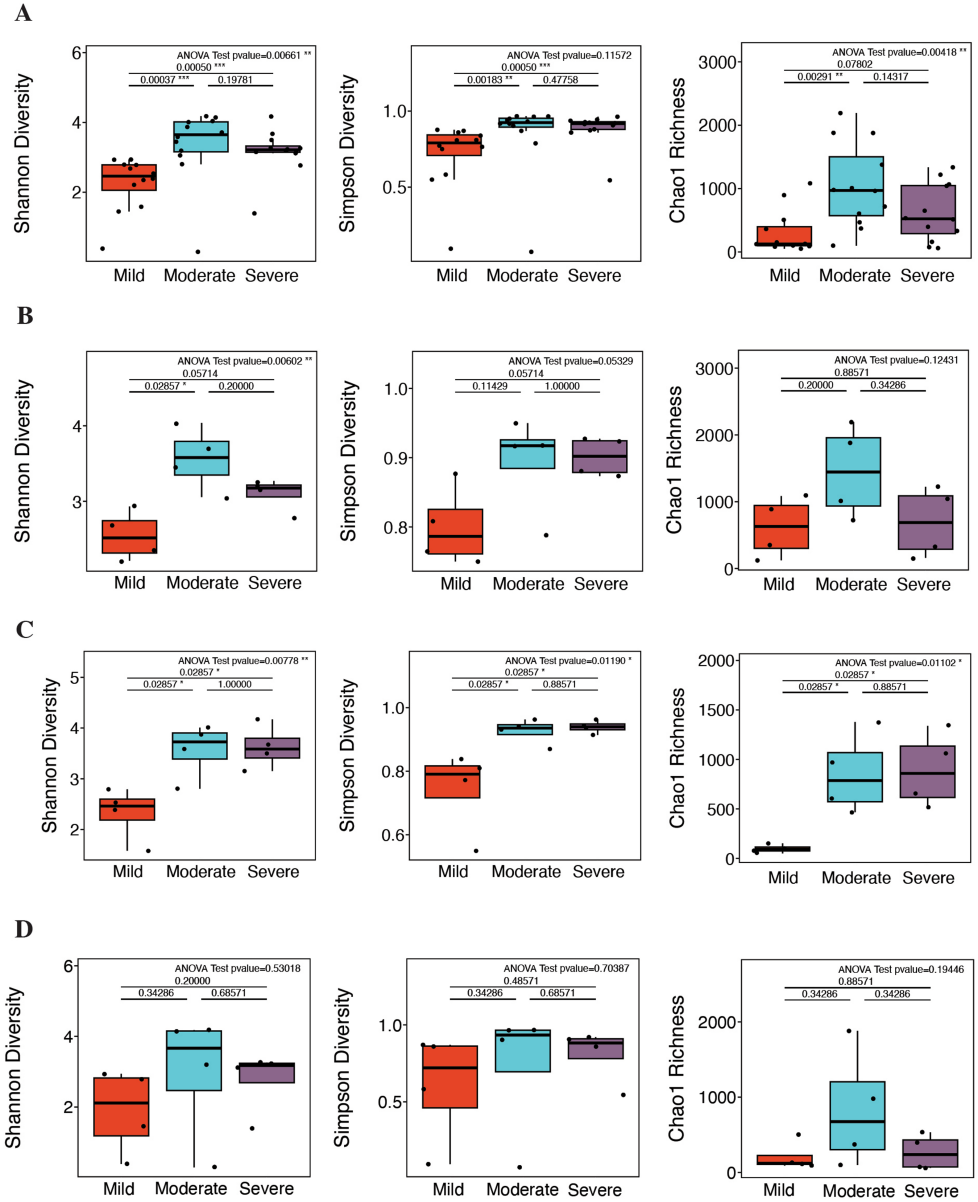
Microbiome diversity analysis in atopic dermatitis (AD) patients across disease severities and anatomical locations. **(A)** Alpha diversity in total samples from AD patients in the mild, moderate, and severe groups. Significant differences were observed in Chao1 and Shannon diversity. **(B)** Alpha diversity in cheek samples from AD patients in the mild, moderate, and severe groups. Significant differences were observed in Shannon diversity. **(C)** Alpha diversity in antecubital samples from AD patients in the mild, moderate, and severe groups. Significant differences were observed in Chao1, Shannon, and Simpson diversity. **(D)** Alpha diversity in popliteal samples from AD patients in the mild, moderate, and severe groups. No significant differences were observed. Asterisks indicate statistical significance (*p < 0.05; **p < 0.01; ***p < 0.001).

Alpha diversity results revealed significant differences in microbial richness and evenness among AD patient groups stratified by disease severity. Specifically, across the entire sample, Shannon diversity and Chao1 richness showed significant differences between mild, moderate, and severe AD groups (*p* = 0.007 and *p* = 0.004, respectively). Overall diversity was lower in the mild group, peaked in the moderate group, and then declined in the severe group, although Simpson diversity in the severe group remained higher than that in the mild group, as shown in Figure 1A. Further analysis of specific sampling sites revealed differences across severity groups. In cheek samples, as shown in Figure 1B, Shannon diversity differed significantly among severity groups (*p* = 0.006), increasing from the mild group to the moderate group and then decreasing in the severe group, with the moderate group showing the highest diversity. In antecubital samples, as shown in Figure 1C, Simpson diversity, Shannon diversity, and Chao1 richness differed significantly among severity groups (*p* = 0.012, *p* = 0.008, and *p* = 0.011, respectively). All three indices were lower in the mild group and higher in the moderate and severe groups, with no significant difference between the moderate and severe groups. In popliteal samples, no significant differences were observed in Shannon diversity, Simpson diversity, or Chao1 richness among severity groups, as depicted in Figure 1D. The moderate group showed a trend toward higher alpha diversity and richness, whereas the mild and severe groups exhibited relatively lower values. Furthermore, as depicted in Supplementary Figure S1, no statistically significant differences were observed in alpha diversity indices between AD patients and healthy controls for the total samples or across the three sampled sites.

**Figure 2:**
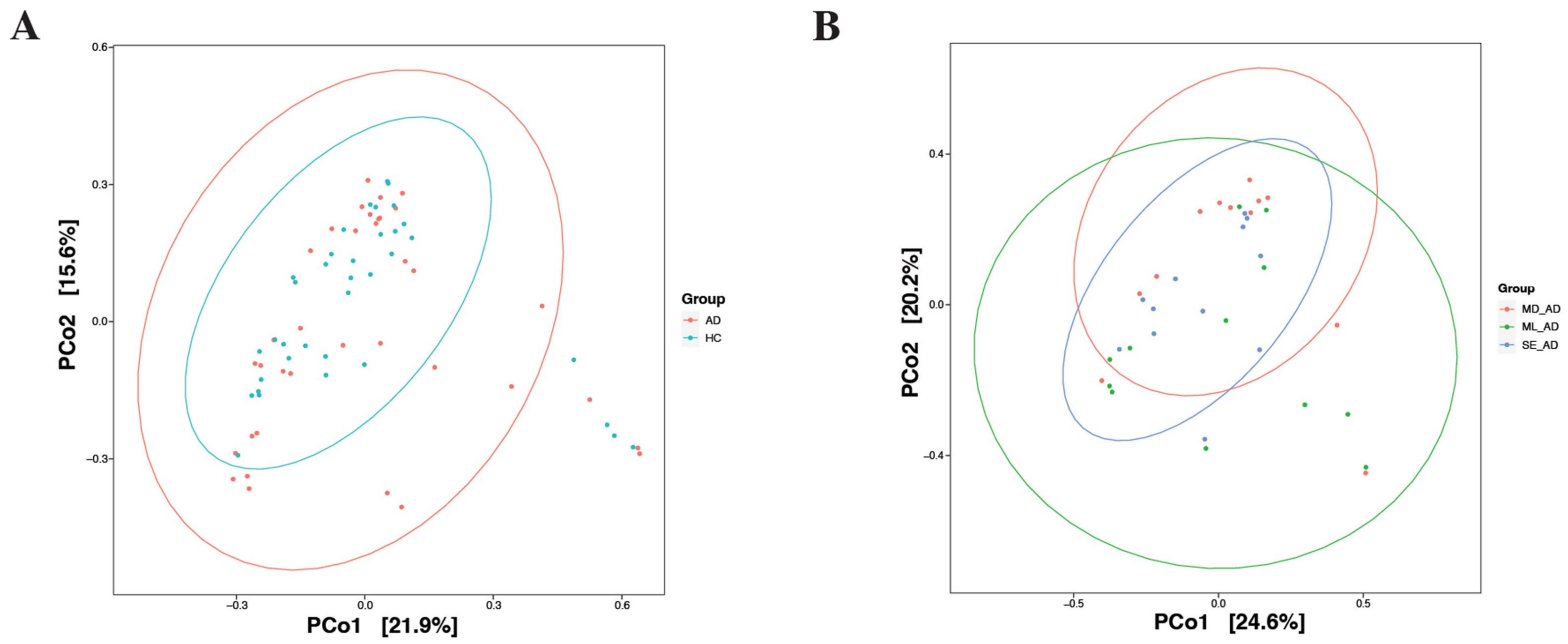
Beta diversity principal coordinate analysis (PCoA) plot comparing **(A)** AD patients to healthy individuals and **(B)** across mild, moderate, and severe groups. The ellipses represent 95% confidence intervals for each group, and individual points represent samples after dimensionality reduction. No clear separation was observed between AD patients and healthy individuals. However, distinct separation was observed between mild and moderate groups, and between moderate and severe groups. AD, atopic dermatitis; HC, healthy control; ML, mild; MD, moderate; SE, severe.

Beta diversity analysis indicated no significant overall separation between AD patients and healthy controls (*p* = 0.187), as shown in Figure 2A. Within the AD cohort, PCoA plots of overall samples displayed partial clustering by severity, with significant differences between mild and moderate groups (*p* = 0.042) and between moderate and severe groups (*p* = 0.036), as shown in Figure 2B. No significant differences were detected in beta diversity among the three sites within the AD or healthy control groups, nor across severity levels at individual sites, as shown in Supplementary Figure S2.

### Taxonomic composition of skin microbiota

**Figure 3:**
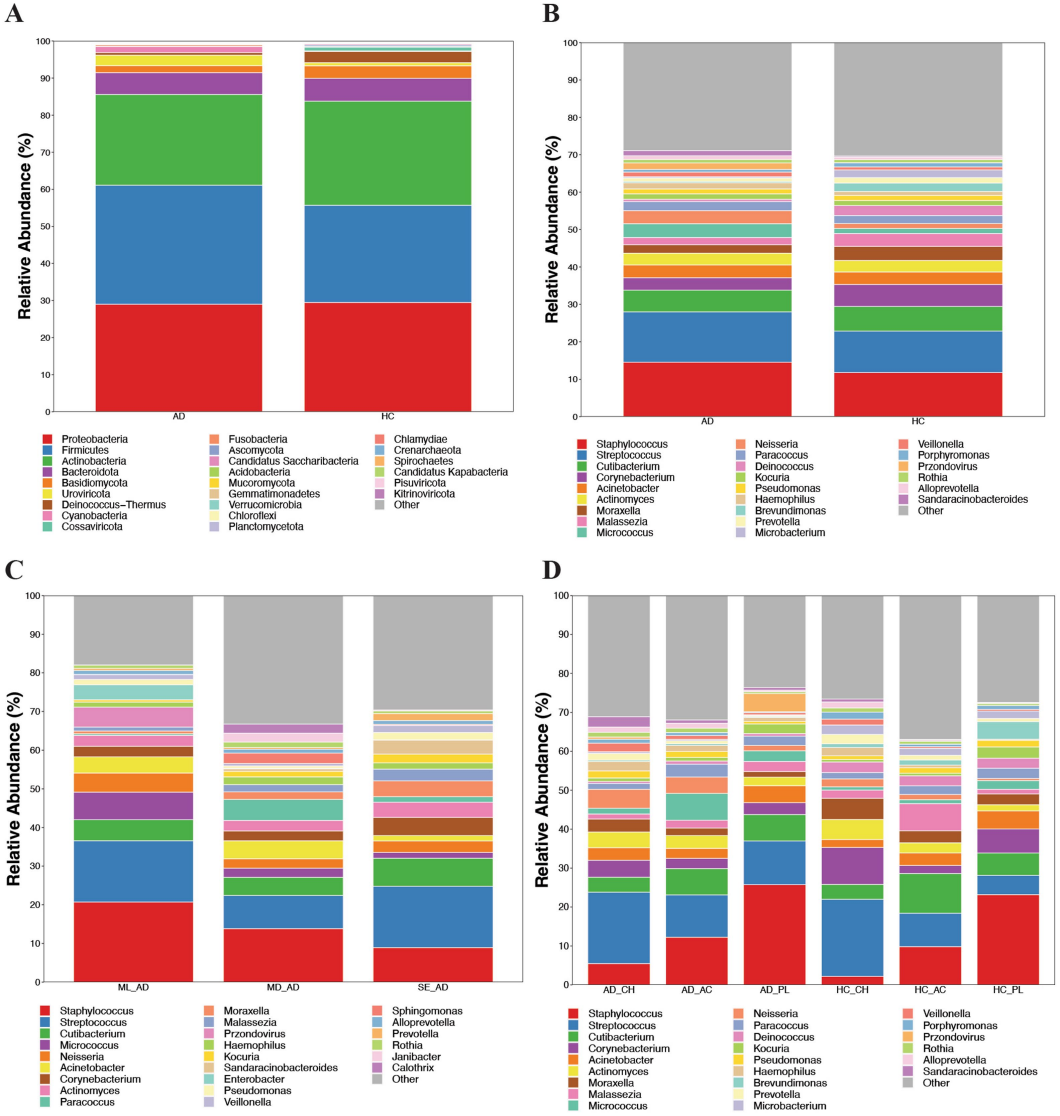
Microbial abundance in patients with atopic dermatitis (AD). **(A)** Microbial abundance at the phylum level in patients with AD compared with healthy controls (HC). *Proteobacteria* was the predominant phylum in patients with AD. **(B)** Microbial abundance at the genus level in patients with AD compared with HC. *Staphylococcus* and *Streptococcus* were the dominant genera in patients with AD. **(C)** Microbial abundance in patients with AD according to disease severity. **(D)** Site-specific microbial abundance in patients with AD and HC. Site-specific subgroup analyses revealed that the relative abundance of *Staphylococcus* in popliteal fossa samples from patients with AD was significantly higher than that in cheek samples (*p* = 0.027). AD, atopic dermatitis; HC, healthy control; ML, mild; MD, moderate; SE, severe; CH, cheek samples; AC, antecubital samples; PL, popliteal samples.

At the phylum level, the overall microbial community structure across all skin samples was dominated by *Proteobacteria* (29.25%), *Firmicutes* (29.15%), and *Actinobacteria* (26.27%), collectively accounting for over 80% of the microbiota, which is depicted in Figure 3A. At the genus level, the top five most abundant genera in AD patients were *Staphylococcus*, *Streptococcus*, *Cutibacterium*, *Micrococcus*, and *Neisseria*, whereas in healthy controls, they were *Staphylococcus*, *Streptococcus*, *Cutibacterium*, *Corynebacterium*, and *Moraxella*. Notably, genera from the *Firmicutes phylum*, particularly *Staphylococcus* and *Streptococcus*, predominated in the AD group, with relative abundances of 14.54% and 13.46% in total AD samples, compared to 11.77% and 11.08% in healthy controls, as shown in Figure 3B and Supplementary Figure S3. A total of 91 genera exhibited relative abundances exceeding 0.1% in the AD group, while 92 such genera were identified in healthy controls. However, no significant differences were observed in the abundances of major genera between AD patients and healthy controls. Furthermore, no significant differences in major microbial abundances were detected across subgroups stratified by disease severity, as depicted in Figure 3C. Site-specific subgroup analyses revealed that the relative abundance of *Staphylococcus* in the popliteal fossa of AD patients was significantly higher than in cheek samples (*p* = 0.027), as shown in Figure 3D.

### Functional profiling of skin microbiota in AD patients

**Figure 4:**
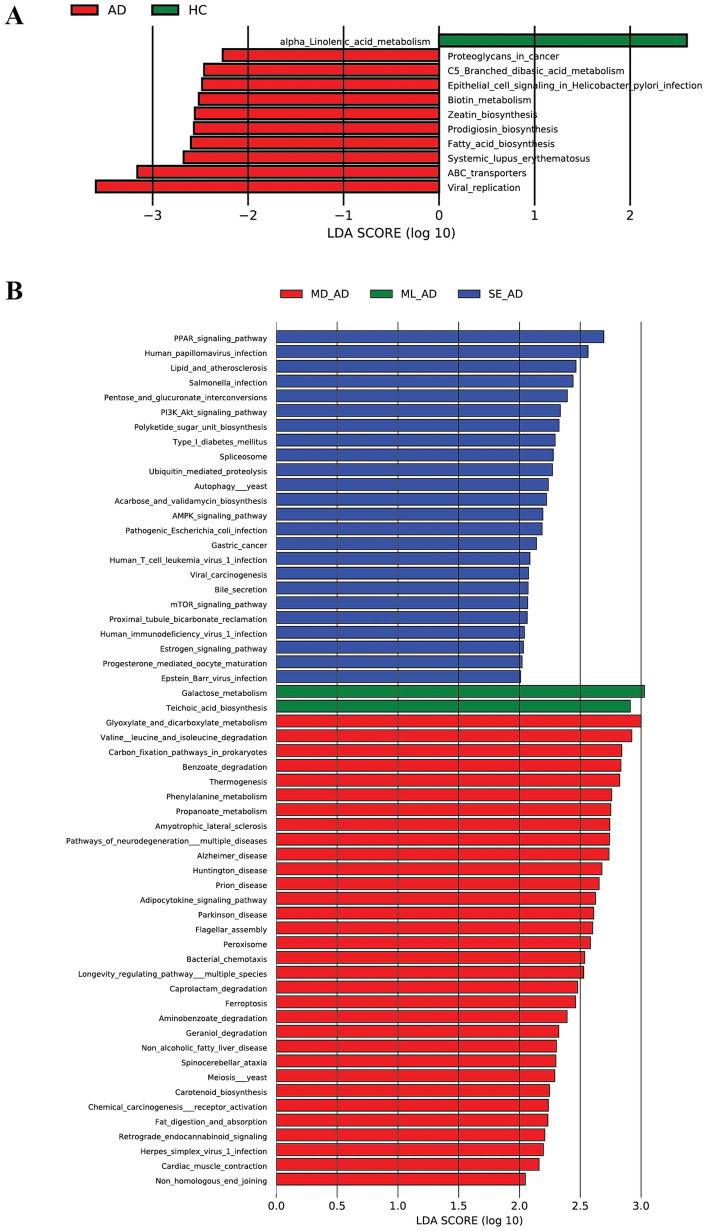
Kyoto encyclopedia of genes and genomes (KEGG) pathway enrichment in samples from patients with atopic dermatitis (AD). **(A)** KEGG pathway enrichment of microbial communities in overall samples from patients with AD compared with healthy controls. Red bars indicate pathways significantly enriched in the AD group, whereas green bars indicate pathways enriched in healthy samples. Ten pathways were significantly enriched in the AD group. **(B)** KEGG pathway enrichment of microbial communities in overall samples across mild, moderate, and severe AD groups. Different colors represent pathways enriched in each severity group. The figure shows that the moderate AD group exhibited the greatest number of enriched pathways overall. AD, atopic dermatitis; HC, healthy control; ML, mild; MD, moderate; SE, severe; KEGG, Kyoto encyclopedia of genes and genomes.

KEGG pathway enrichment analysis revealed differentially enriched pathways associated with skin microbiota in AD patients compared to healthy controls. In the total samples, 11 pathways were enriched between AD and control groups, with 10 pathways significantly enriched in AD patients, including viral replication, ABC transporters, systemic lupus erythematosus, fatty acid biosynthesis, and prodigiosin biosynthesis, as shown in Figure 4A. Stratified by disease severity in total samples, mild AD patients showed enrichment in only two pathways: galactose metabolism and teichoic acid biosynthesis. Moderate AD patients exhibited enrichment in 32 pathways, such as valine, leucine, and isoleucine degradation. Severe AD patients had 24 enriched pathways, including PPAR signaling pathway and human papillomavirus infection, as shown in Figure 4B. A more detailed KEGG enrichment analysis of samples from different patient sites and sites with varying severity levels is presented in Supplementary Figures S4–S5.

**Figure 5:**
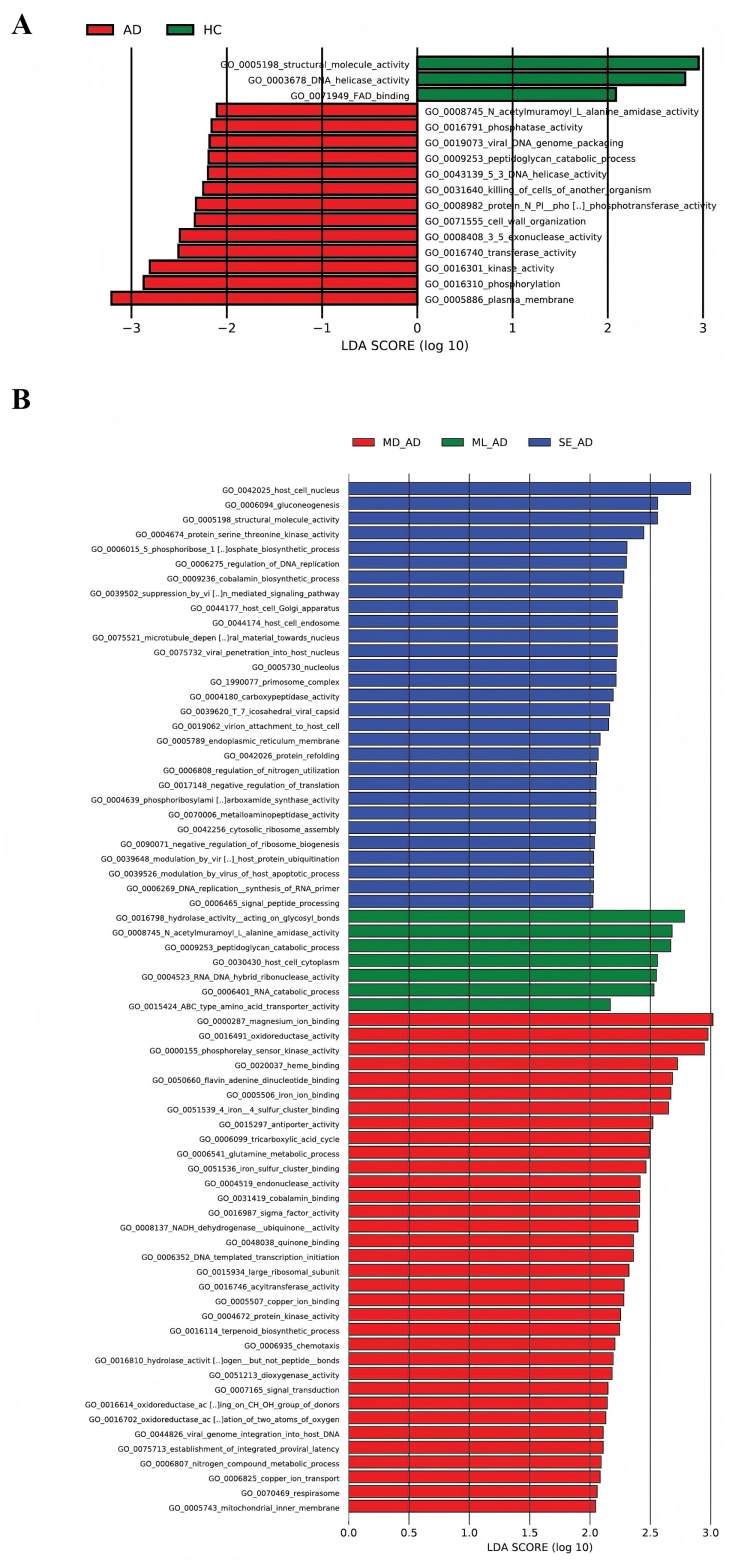
Gene ontology (GO) enrichment in samples from patients with atopic dermatitis (AD). **(A)** GO enrichment of microbial communities in overall samples from patients with AD compared with healthy controls. Red bars indicate terms significantly enriched in the AD group, whereas green bars indicate terms enriched in healthy samples. Thirteen terms were significantly enriched in the AD group. **(B)** GO enrichment of microbial communities in overall samples across mild, moderate, and severe AD groups. Different colors represent terms enriched in each severity group. The figure shows that the moderate AD group exhibited the greatest number of enriched terms overall, with 34 terms identified. AD, atopic dermatitis; HC, healthy control; ML, mild; MD, moderate; SE, severe; GO, gene ontology.

Furthermore, a total of 16 GO terms were enriched in the total samples, with 13 enriched in the AD group. The majority of differentially abundant genes were found to be enriched in functions related to the plasma membrane, phosphorylation, and various enzymatic activities such as kinase activity, transferase activity, 3′-5′ exonuclease activity, and phosphatase activity. Terms involved in cell wall organization, peptidoglycan catabolism and N-acetylmuramoyl-L-alanine amidase activity were also significantly enriched in the AD cohort, as shown in Figure 5A. Additionally, as illustrated in Figure 5B, skin microbial samples from mild, moderate, and severe AD patients enriched 7, 34, and 29 GO terms, respectively. The results of the GO enrichment analysis for samples from different patient sites and sites with varying severity levels are provided in Supplementary Figures S6–S7.

Virulence factors were annotated in 30 samples overall, with 26 enriched in AD samples. These included genes related to *Staphylococcus* capsules, toxins like *γ*-hemolysin, adhesins like AdsA, and secretion systems such as the type III and type VI secretion system, as shown in Supplementary Figure S8A. Additionally, the three AD severity samples were annotated with 2, 34, and 12 virulence factors, respectively, as shown in Supplementary Figure S8B. The results of virulence factor analysis for samples from different patient sites and sites with different severity levels are shown in Supplementary Figures S9–S10.

PHI analysis of the total sample enriched 49 genes. Among these, the AD group enriched genes including *argF*, *PitA*, *arcA*, and several toxin-related genes such as *hlgB* and *lukE*, as shown in Supplementary Figure S11A. Additionally, as depicted in Supplementary Figure S11B, 6 genes including *GyrA*, 99 genes including *pstS* and *kdpB*, and 39 genes including *CspA* and *esxA1* were enriched in mild, moderate, and severe AD patient samples, respectively. The results of PHI analysis for samples from different sites and varying degrees of severity in the patient are presented in Supplementary Figures S12–S13.

## Discussion

In this study, metagenomic approaches were used to characterize differences in the microbial composition and functional profiles of skin lesions in pediatric patients with AD. Overall, *Staphylococcus* and *Streptococcus* were the most abundant bacterial genera in children with AD. Although increased detection of *Staphylococcus* and *Streptococcus* in AD lesions has been reported previously, our findings extend the current understanding in several ways. First, in this pediatric cohort, overall abundance alone did not distinguish severity groups, whereas site-stratified analysis revealed significantly higher *Staphylococcus* abundance in the popliteal fossa than in cheek lesions, indicating a location-dependent microbial pattern. Second, alpha-diversity varied nonlinearly across severity groups, with the highest diversity observed in moderate AD, suggesting that microbial dynamics in pediatric AD may differ from the monotonic decline often reported in adults. Together, these findings suggest that, in children with AD, lesion location and disease stage may shape the skin microbiome in a manner not evident from all lesions.

Several other genera, including *Corynebacterium*, *Micrococcus*, and *Neisseria*, were also among the five most abundant genera in the AD group. *Corynebacterium* was detected in both AD and healthy control samples, and no significant overall reduction was observed, suggesting that AD may have a limited effect on its abundance in pediatric skin. As a common skin-associated commensal genus, *Corynebacterium* may also contribute to microbial homeostasis, although its role in AD appears to be context-dependent (24, 25). In addition, previous studies have suggested that *Micrococcus* may enhance the virulence of *S. aureus*, whereas *Neisseria* may be involved in oral-skin microbial transfer in AD (26, 27). Although these findings require further validation, their presence in our cohort suggests that microbial interactions beyond *Staphylococcus* may also contribute to the pediatric AD microbiome.

Notably, the alpha-diversity pattern observed across severity groups may reflect dynamic microbial changes during disease progression. In adult AD, lower alpha diversity is often associated with increasing severity (28). However, our pediatric samples in this study showed the highest diversity and richness in the moderate group. In the severe group, the decline in Chao1 richness may indicate a reduction in species number, although the differences between the moderate and severe groups were not statistically significant. This nonlinear relationship, together with the beta-diversity pattern showing severity-related clustering within the AD cohort, may suggest the existence of a transitional microbial state in pediatric AD. Supporting this interpretation, a previous study identified distinct and temporally stable microbial dermotypes associated with disease severity, raising the possibility that AD progression involves shifts between alternative microbiome states (29). Meanwhile, the alpha diversity of the popliteal fossa in pediatric patients with AD showed greater variability than that of other body sites, suggesting larger inter-individual differences in microbial diversity at this specific site in children.

Functional analysis of the skin microbiome in pediatric AD patients suggests that microbial activity changes occur during the onset and progression of AD. KEGG pathways enriched in AD patients, such as ABC transporters, fatty acid biosynthesis, and prodigiosin biosynthesis, along with GO terms including kinase activity, transferase, 3′ to 5′ exonuclease, plasma membrane, and phosphorylation, suggest microbial functions related to replication, repair, and recombination, as well as pathogenicity and survival (30–33). In particular, fatty acid biosynthesis may be mechanistically relevant to AD because epidermal lipid-barrier disruption favors staphylococcal overgrowth, while staphylococcal fatty-acid metabolic remodeling has been linked to membrane adaptation, protease regulation, and enhanced virulence during skin infection (34, 35). Likewise, the enrichment of kinase activity and phosphorylation is consistent with bacterial two-component regulatory systems that control toxins, proteases, and other virulence determinants, whereas ABC transporters may further facilitate persistence on AD skin by mediating antimicrobial-peptide resistance and virulence-associated peptide export (36, 37). Broad categories such as transferase activity, 3′-to-5′ exonuclease, and prodigiosin biosynthesis are more cautiously interpreted as general metabolic or survival-associated functions and direct evidence linking them specifically to AD pathogenesis remains limited.

The enriched terms specific to each body site reflect the microbial activity characteristics at corresponding locations, potentially indicating distinct microbial profiles among AD patients across different sites. First, the primary enriched terms identified across the three sampling sites correspond to common microbial metabolic processes, including antioxidant stress response, genome replication and repair, and protein synthesis (38–40). However, subtle differences still exist. The enrichment of adipokine signaling pathways in the cheeks of severe patients may reflect metabolic and inflammatory dysregulation. A previous study indicated that adipokines involved in this pathway are often imbalanced in AD patients and correlate with disease severity (41). The enrichment of heme-binding pathways in the cheeks and popliteal fossa lesions of moderate AD patients likely indicates bacterial activity, such as that of *S. aureus*, which can utilize the iron-responsive surface regulator system to acquire heme iron for growth (42). The enrichment of flagellar assembly genes in the elbow crease suggests heightened activity of gut-derived bacteria in this region (43, 44). This variation may indicate a degree of heterogeneity in AD across different body sites. In severe AD subgroups, specific sites such as the face show enrichment of the adipocytokine signaling pathway, suggesting altered adipokine-related signaling that may contribute to local immune dysregulation. Adipokines have been implicated in immune imbalance in obese patients with AD (45). However, the relevance of this pathway to site-specific microbial interactions requires further validation.

Virulence factors and PHI analysis suggest the potential impact of the microbiome on skin lesions in AD patients. First, the enrichment of pathogenic factors such as *Staphylococcus* capsules and *γ*-hemolysin, along with PHI genes like *hlgB* and *lukE* in AD patients, indicates these factors both promote bacterial survival and disrupt the skin barrier (46–48). The enrichment of the virulence factor AdsA and the antibiotic resistance-associated PHI gene *arcA* in AD patients further suggests that bacteria such as *S. aureus* may exhibit enhanced survival capabilities within AD lesions (49, 50). Furthermore, the shift in predominant virulence factors from superantigens such as *Spes* in mild AD to lipopolysaccharides derived from *Pseudomonas* in moderate-to-severe cases suggests that Gram-negative bacteria may contribute to disease progression and exacerbation (51, 52). Naturally, these conclusions require further experimental validation.

This study has several limitations. A relatively small sample size and single-center recruitment may have introduced geographical bias and limited the generalizability of the findings. Moreover, despite controlling baseline characteristics as much as possible, the use of skin samples from pediatric patients with AD remains a limitation, as age-related variation and environmental exposures from children’s play behaviors or living environment before sampling may influence skin microbiome abundance and diversity. Furthermore, although shotgun metagenomic sequencing can provide species-level taxonomic information, the present study focused primarily on genus-level microbial patterns, as species-level assignments may be less robust in skin samples with low microbial biomass and a high proportion of host-derived DNA, particularly for low-abundance or closely related taxa. Finally, translating metagenomic sequencing into routine clinical practice remains challenging. The microbial composition on individual skin surfaces is highly susceptible to environmental exposure and lifestyle factors, potentially hindering the reproducibility of test results and clinical applicability.

## Conclusion

In conclusion, this study demonstrates that the composition, diversity, and functional characteristics of skin microbiome in children with AD exhibit complex alterations compared to healthy controls. Moderate and severe pediatric AD patients show distinct microbiome diversity. Additionally, the abundance of *Staphylococcus* was significantly higher in the popliteal fossa compared to the cheeks and antecubital fossa. Functional analysis revealed enrichment of metabolic pathways, pathogenic factors, and PHI genes in the epidermal microbiota of pediatric AD patients, suggesting dysbiosis accompanied by altered microbial activities, potentially site- and severity-specific. These findings warrant further validation.

## Data Availability

The data presented in the study are deposited in the NCBI BioProject repository (https://www.ncbi.nlm.nih.gov/bioproject/PRJNA1256207), accession number PRJNA1256207.
