# Supplementary material for: Distinct microbial and functional alterations across skin sites and disease severity in pediatric atopic dermatitis: a prospective study
Source: Front Med (Lausanne). 2026 Apr 29;13:1805596. doi: 10.3389/fmed.2026.1805596 (PMC13168024; doi:10.3389/fmed.2026.1805596)
Supplement: Supplementary file 1 [file Supplementary_file_1.docx]

Supplementary Materials

Supplementary Materials for Distinct Microbial and Functional Alterations Across Skin Sites and Disease Severity in Pediatric Atopic Dermatitis

# Supplementary Material

## A1 Kyoto Encyclopedia of Genes and Genomes analysis results for the subgroup of body regions

Site-specific Kyoto Encyclopedia of Genes and Genomes (KEGG) analyses revealed distinct patterns. In cheek samples from atopic dermatitis (AD) and control groups, four pathways were enriched overall, with pentose phosphate pathway and biotin metabolism significantly enriched in AD. Antecubital samples showed nine enriched pathways, seven of which were significantly enriched in AD, including aminoacyl-tRNA biosynthesis and thiamine metabolism. Popliteal samples had four enriched pathways, with MAPK signaling pathway significantly enriched in AD patients.

Further stratification by disease severity and sampling site revealed distinct enrichment patterns. In cheek samples, mild AD was associated with four enriched pathways such as phosphotransferase system, moderate AD with 11 pathways such as carbon metabolism, and severe AD with three pathways such as adipocytokine signaling pathway. For antecubital samples, mild AD featured three enriched pathways including biosynthesis of secondary metabolites, moderate AD included 19 pathways such as flagellar assembly, and severe AD encompassed 41 pathways including Alzheimer disease. In popliteal samples, no enriched pathways were identified in mild AD, whereas moderate AD showed eight enriched pathways such as valine, leucine, and isoleucine degradation, and severe AD displayed five pathways such as mismatch repair.

## A2 Gene Ontology analysis results for the subgroup of body regions

Gene Ontology (GO) analysis of different anatomical sites revealed localized functional alterations. In cheek samples from AD patients, two GO terms were enriched, including methyltransferase activity. Antecubital fossa samples exhibited a more pronounced difference with 19 significantly enriched terms, prominently featuring functions associated with the GTP binding. In the popliteal region, the AD group showed significant enrichment in two gene functions, including DNA-directed DNA polymerase activity.

A more detailed analysis stratified by severity and body region provides further insights. In cheek samples, the number of significantly enriched terms progressed from one in mild AD, related to carbohydrate derivative metabolic processes, to six in moderate AD, including heme binding, and four in severe AD, such as ribonuclease activity. The antecubital fossa displayed the most substantial functional enrichment, with 21 terms like RNA binding enriched in mild cases, 53 terms including cell outer membrane functions in moderate cases, and 18 terms such as acyltransferase activity in severe cases. Finally, in the popliteal fossa, mild AD was characterized by two significantly enriched terms, one of which was N-acetylmuramoyl-L-alanine amidase activity. Both moderate and severe popliteal AD featured 13 significantly enriched terms, with notable functions including heme binding in moderate AD and translation elongation factor activity in severe AD.

## A3 Virulence factor enrichment result for the subgroup of body regions

Site-specific analysis revealed that 6 virulence factors were significantly enriched in AD cheek samples, while 24 factors were enriched in antecubital samples, and 1 factor was significantly enriched in the popliteal samples. The virulence factors that were enriched in the cheek samples included Staphylococcal superantigen-like proteins (SSLs), extracellular adherence protein/MHC analogous protein (Eap/Map), and the XCP secretion system. Moreover, virulence factors enriched in the antecubital samples included alginate from *Pseudomonas*, capsule from *Staphylococcus* and *Streptococcus*. The popliteal samples exhibited enrichment of virulence factors, including SodB from *Legionella*.

In the context of site-specific severity subgroups, the resultant findings are more intricate. In cheek samples, mild AD exhibited five significantly enriched factors, including clumping factor from *Staphylococcus* and HSI-2 T6SS secreted effectors from *Pseudomonas*. Moderate AD manifested nine factors, including type IV pili (TFP) from *Acinetobacter* and flagella from *Burkholderia*. Severe AD displayed three factors, including AhpC from *Mycobacterium*. The antecubital fossa exhibited the most substantial alterations, with enrichment increasing from 1 factor C5a peptidase from *Streptococcus* in mild AD to 57 factors like AdeFGH efflux pump from *Acinetobacter* in moderate AD and remaining high with 36 factors including β-haemolysin from *Streptococcus* in severe AD. Conversely, popliteal samples demonstrated a more constrained response, exhibiting one factor TFP from *Legionella* in mild AD, three factors including ShdA from *Salmonella* in moderate AD, and one factor PcaA from *Mycobacterium* in severe AD.

## A4 Pathogen host interactions enrichment results for the subgroup of body regions

Site-specific pathogen-host interaction analysis revealed the presence of 20 genes in cheek samples, including *tsr*, *proA*, and *cheM*; 56 genes in antecubital samples, including *PstB*, *agrA*, and *Agr*; and 72 genes in popliteal samples, including *acs*, *csrA*, and *purA*, from the AD patient group.

A more thorough examination of site-specific severity subgroups revealed that the number of enriched genes varied among different subgroups.In cheek samples, the number of enriched genes varied substantially across severity groups: only 1 gene (*yegN*) was enriched in mild AD, increasing to 18 genes in moderate AD (including *ompA*, *copA*, and *hopA1*), and then decreasing to 4 genes in severe AD (including *nqrE* and *rmlA*). Antecubital fossa samples showed no significant PHI gene enrichment in mild AD. In contrast, moderate AD was characterized by substantial enrichment of 67 genes (e.g., *clpB*, *gyrA*, *acs*), while severe AD displayed the highest enrichment, with 86 genes (e.g., *covR*, *pvdL*, *stk*). In popliteal fossa samples, no notable PHI gene enrichment was observed in either mild or severe AD. However, moderate AD showed enrichment of 30 genes, including *copA*, *lpxA*, and *tsr*.

# Supplementary Figures


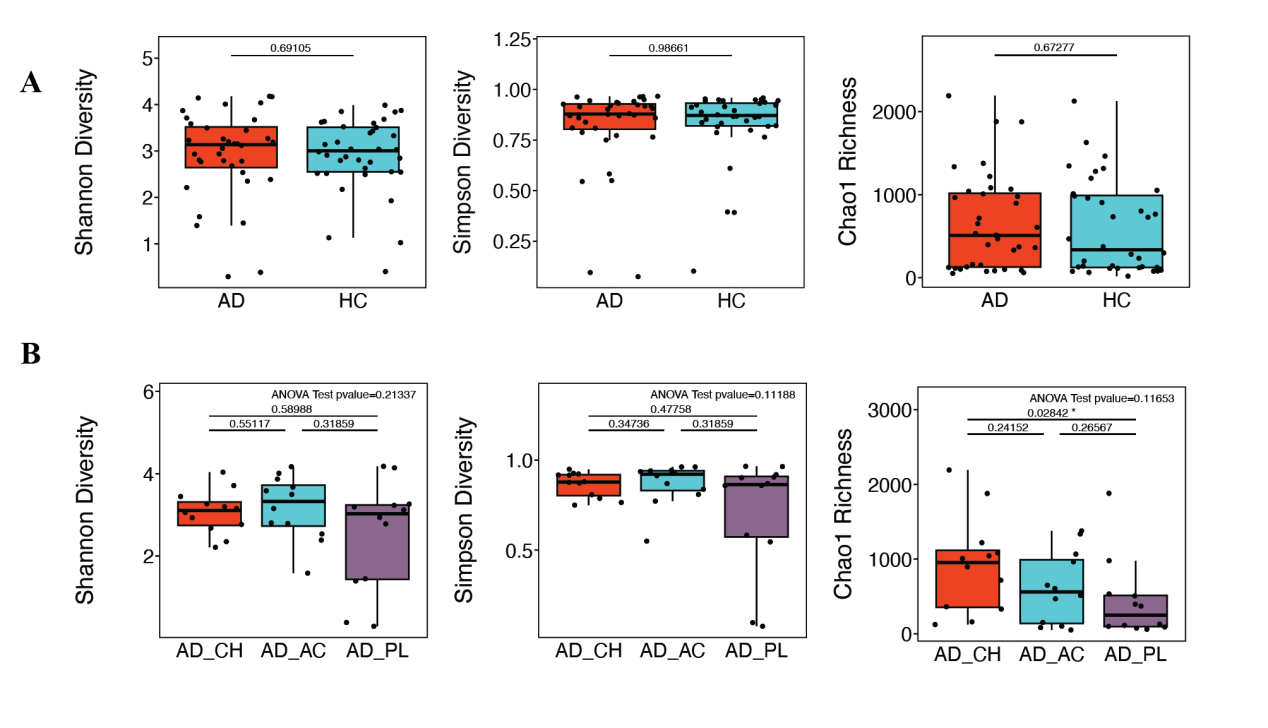


Figure S1. Alpha diversity of (A) the overall samples from atopic dermatitis (AD) patients and healthy controls and (B) across the three sampled sites. As shown, the Chao1, Shannon, and Simpson indices showed no significant differences. AD, atopic dermatitis; HC, healthy control; CH, cheek samples; AC, antecubital samples; PL, popliteal samples.


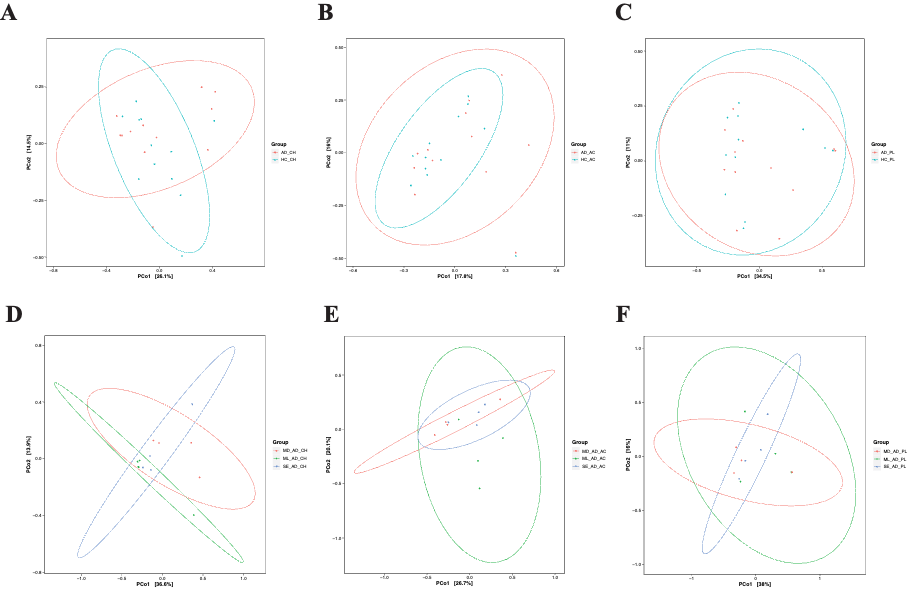


Figure S2. Principal coordinates analysis plots of beta diversity for overall samples stratified by disease severity and for lesion sites stratified by severity. (A-C) represent mild, moderate, and severe cases, respectively. (D-F) represent samples from the cheek, antecubital fossa, and popliteal fossa, respectively, where different colored dots indicate different severity levels. The confidence ellipses in each plot indicate that the groups did not separate significantly. AD, atopic dermatitis; HC, healthy control; ML, mild; MD, moderate; SE, severe; CH, cheek samples; AC, antecubital samples; PL, popliteal samples.


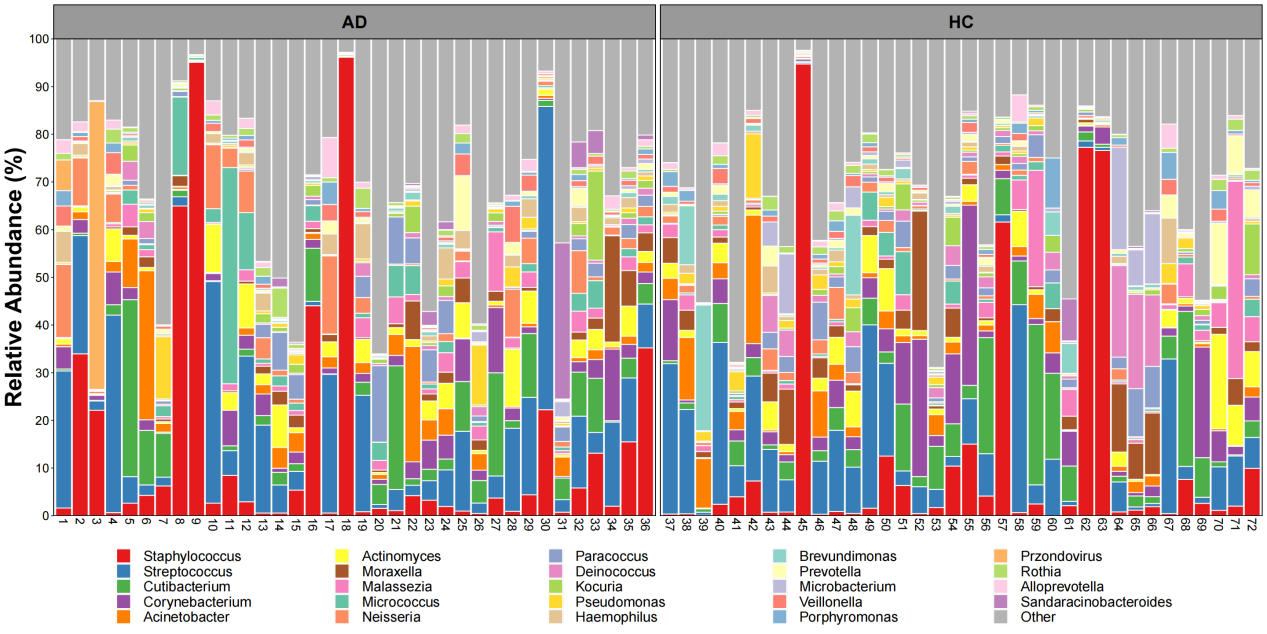


Figure S3. Genus-level microbial composition in individual samples from the AD and HC groups. Stacked bar plots show the relative abundance (%) of the major genera detected in each sample. Each bar represents one individual sample, with samples grouped into AD and HC cohorts as indicated above the panels.


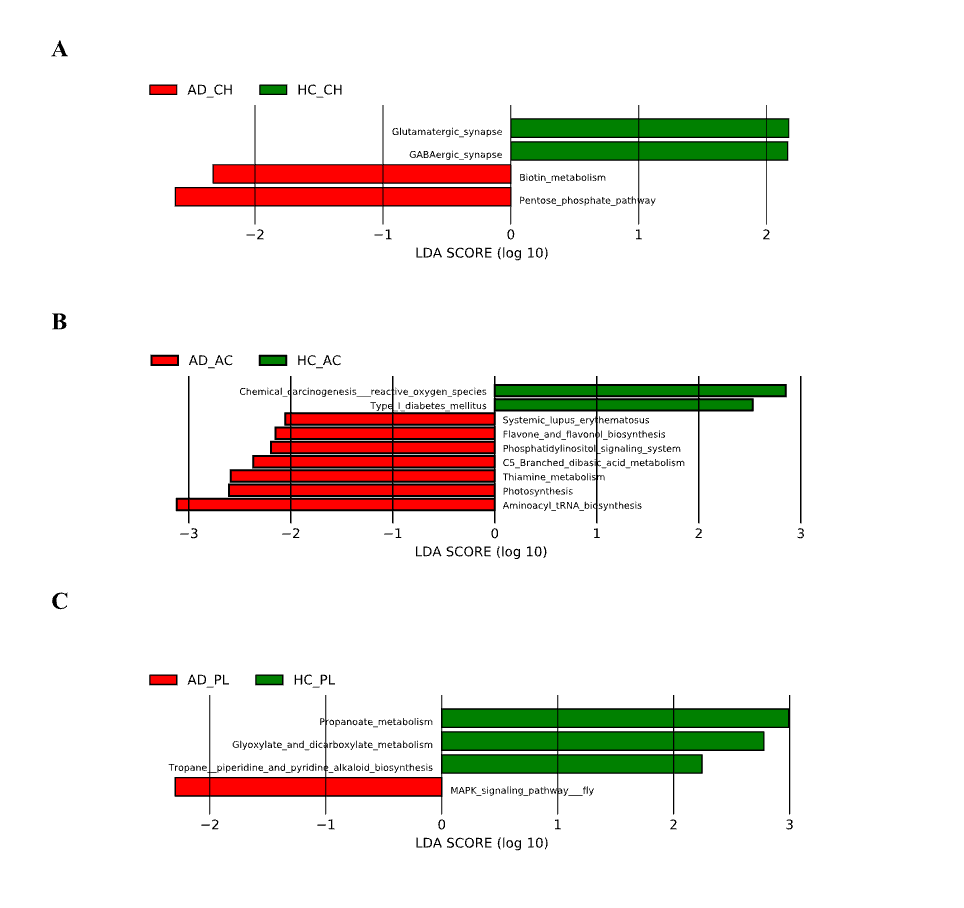


Figure S4. Kyoto Encyclopedia of Genes and Genomes (KEGG) enrichment analysis of samples from the (A) cheek, (B) antecubital fossa, and (C) popliteal fossa in patients with atopic dermatitis (AD). The height of the bars corresponds to the magnitude of difference in enriched features between groups. Red bars represent enrichment in the AD group, and green bars represent enrichment in the healthy control group. AD, atopic dermatitis; HC, healthy control; CH, cheek samples; AC, antecubital samples; PL, popliteal samples.


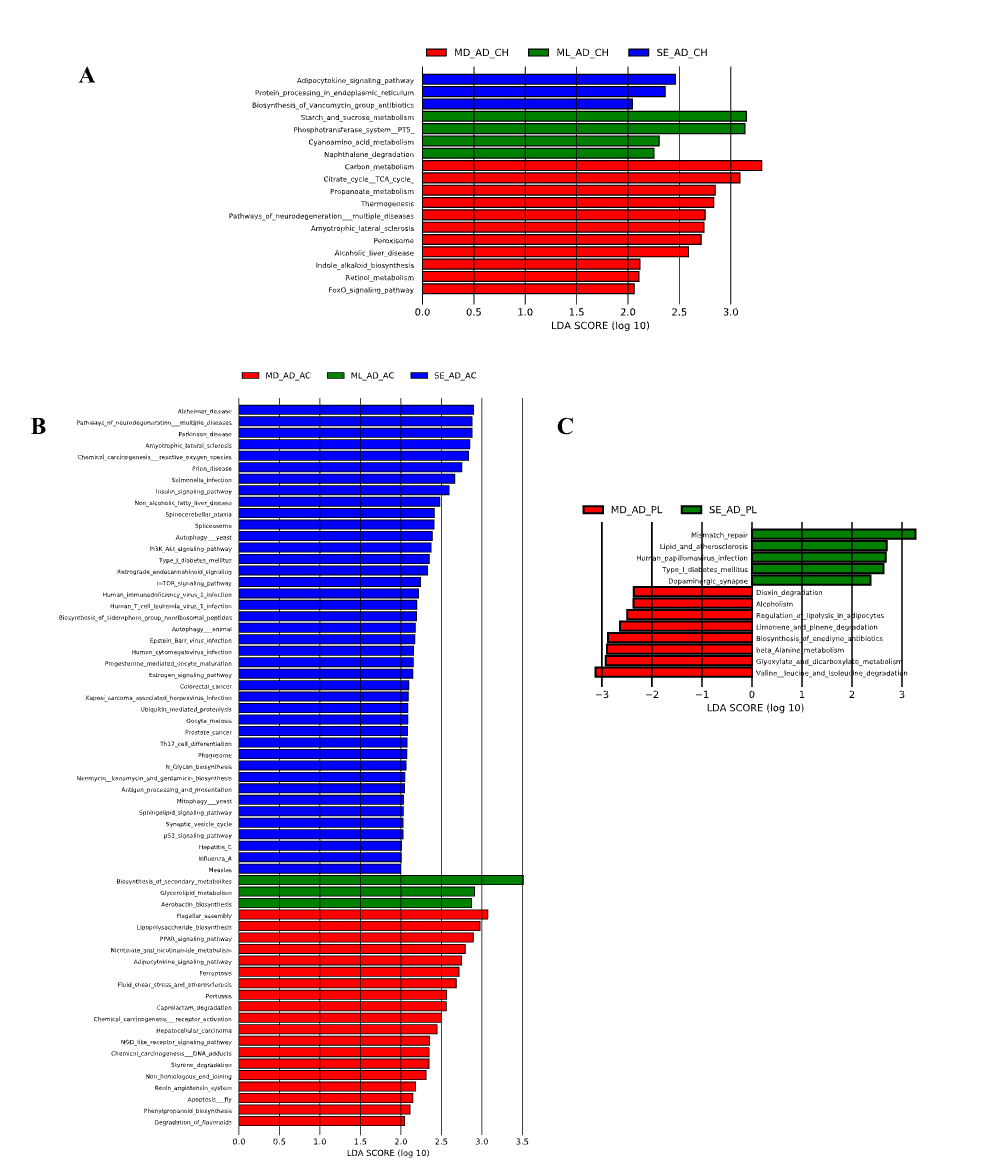


Figure S5. Kyoto Encyclopedia of Genes and Genomes (KEGG) enrichment analysis comparing different disease severities in samples from (A) the cheek, (B) the antecubital fossa, and (C) the popliteal fossa of patients with atopic dermatitis (AD). The bar chart represents the enrichment status. A higher bar indicates a greater difference in the enriched features between the compared group. AD, atopic dermatitis; HC, healthy control; ML, mild; MD, moderate; SE, severe; CH, cheek samples; AC, antecubital samples; PL, popliteal samples.


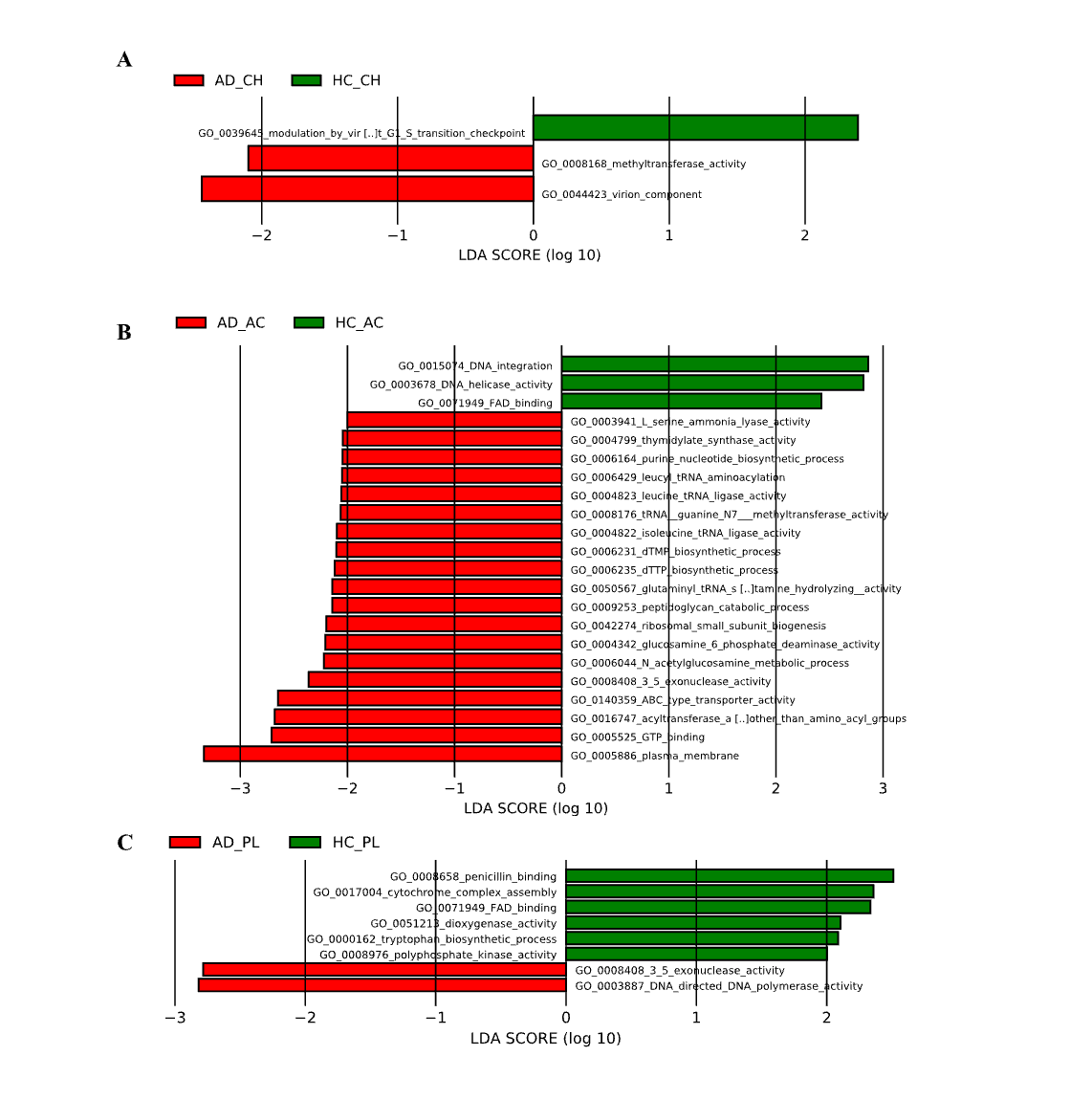


Figure S6. Gene Ontology (GO) enrichment analysis in samples from (A) the cheek, (B) the antecubital fossa, and (C) the popliteal fossa of atopic dermatitis (AD) patients compared to healthy controls. The bar chart represents the enrichment status; a higher bar indicates a greater difference in the enriched features between the compared groups. AD, atopic dermatitis; HC, healthy control; CH, cheek samples; AC, antecubital samples; PL, popliteal samples.


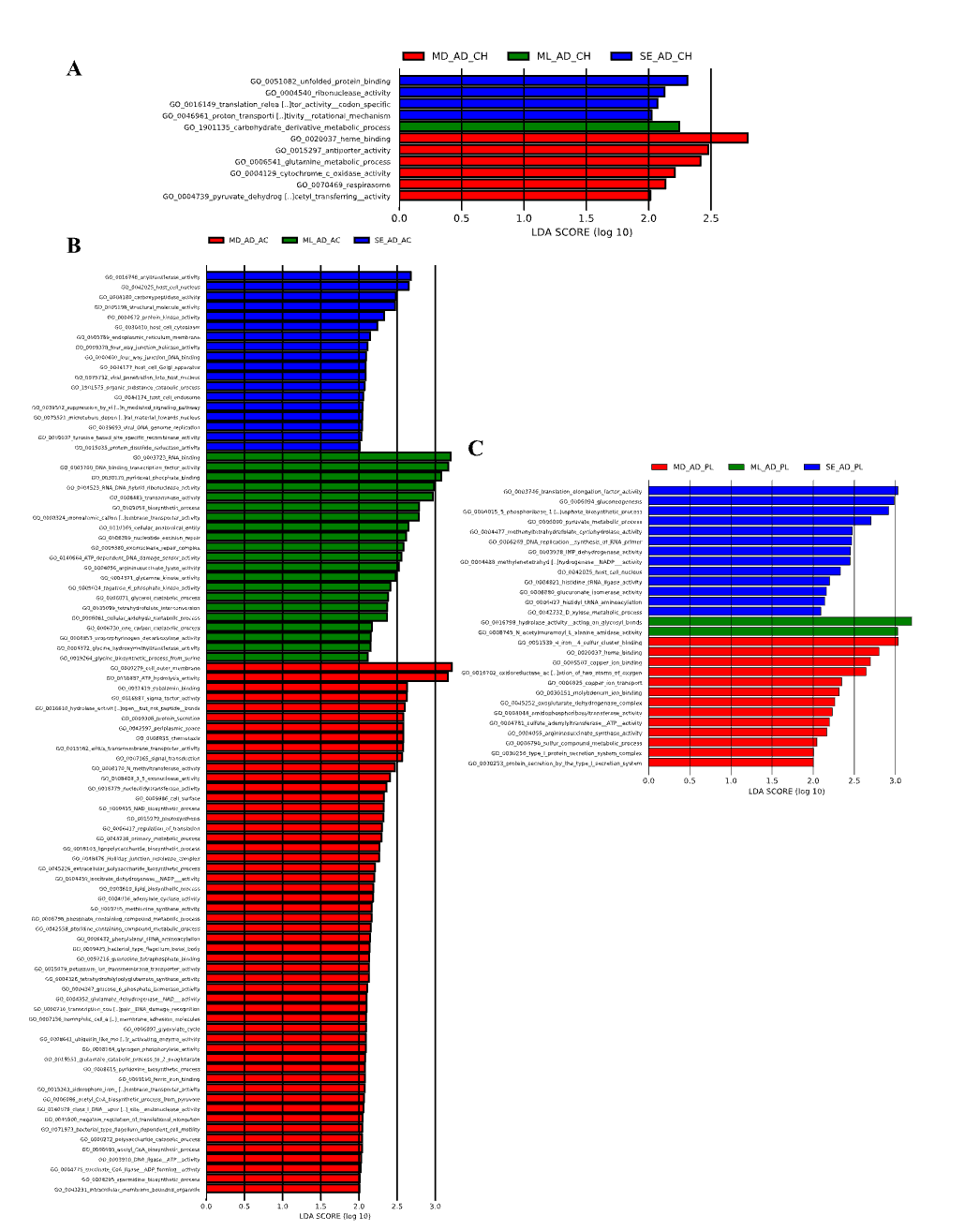


Figure S7. Gene Ontology (GO) enrichment analysis comparing different disease severities in samples from (A) the cheek, (B) the antecubital fossa, and (C) the popliteal fossa of patients with atopic dermatitis (AD). The bar chart represents the enrichment status; a higher bar indicates a greater difference in the enriched features between the compared groups. AD, atopic dermatitis; HC, healthy control; ML, mild; MD, moderate; SE, severe; CH, cheek samples; AC, antecubital samples; PL, popliteal samples.


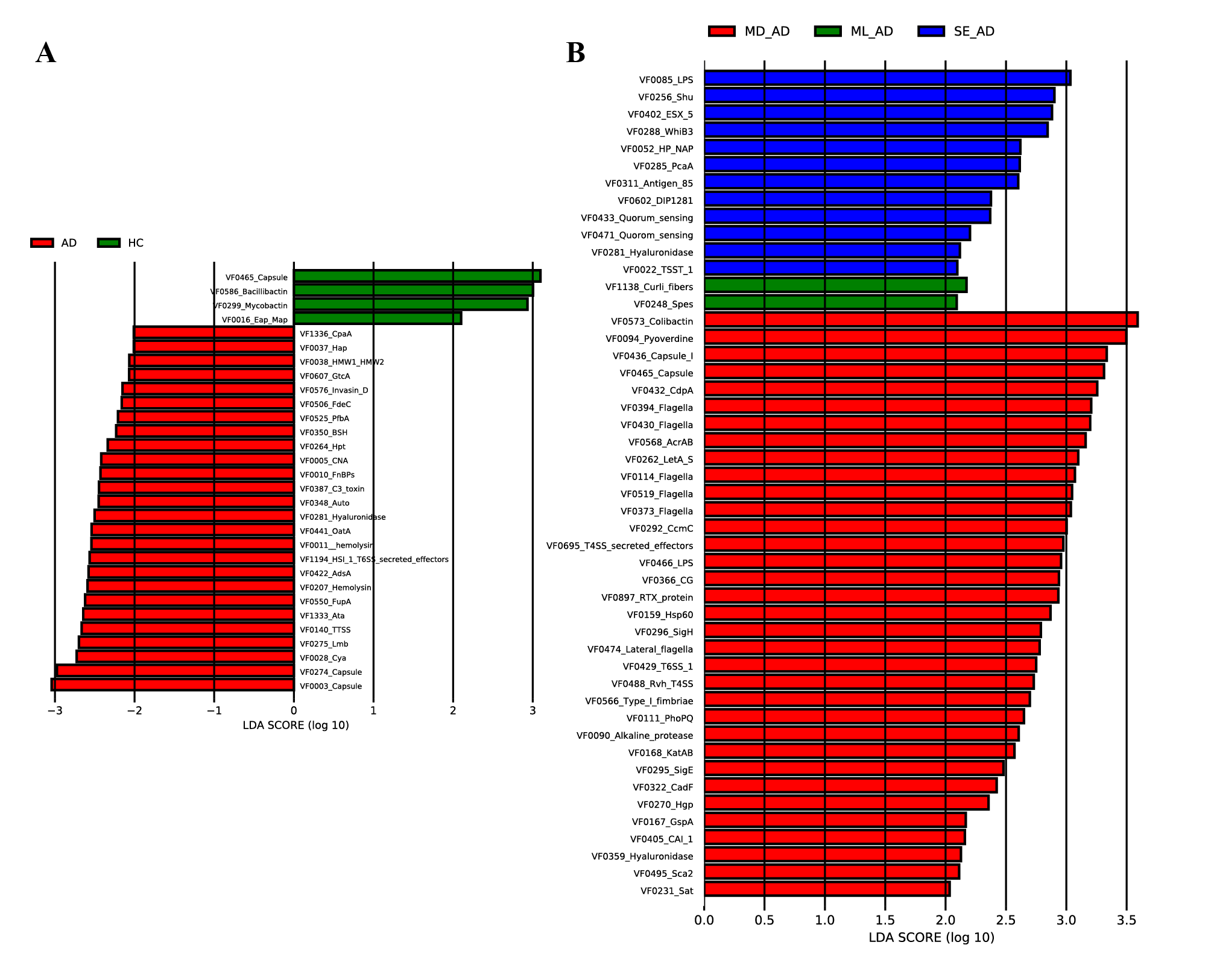


Figure S8. Enrichment of virulence factors in samples from patients with atopic dermatitis (AD). (A) Enrichment of microbial virulence factors in overall samples from patients with AD compared with healthy controls. Red bars indicate virulence factors significantly enriched in the AD group, whereas green bars indicate those enriched in healthy samples. Twenty-six virulence factors were significantly enriched in the AD group. (B) Enrichment of microbial virulence factors in overall samples across mild, moderate, and severe AD groups. Different colors represent virulence factors enriched in each severity group. The figure shows that the moderate AD group exhibited the greatest number of enriched virulence factors overall, with 34 in total. AD, atopic dermatitis; HC, healthy control; ML, mild; MD, moderate; SE, severe. VF, virulence factor.


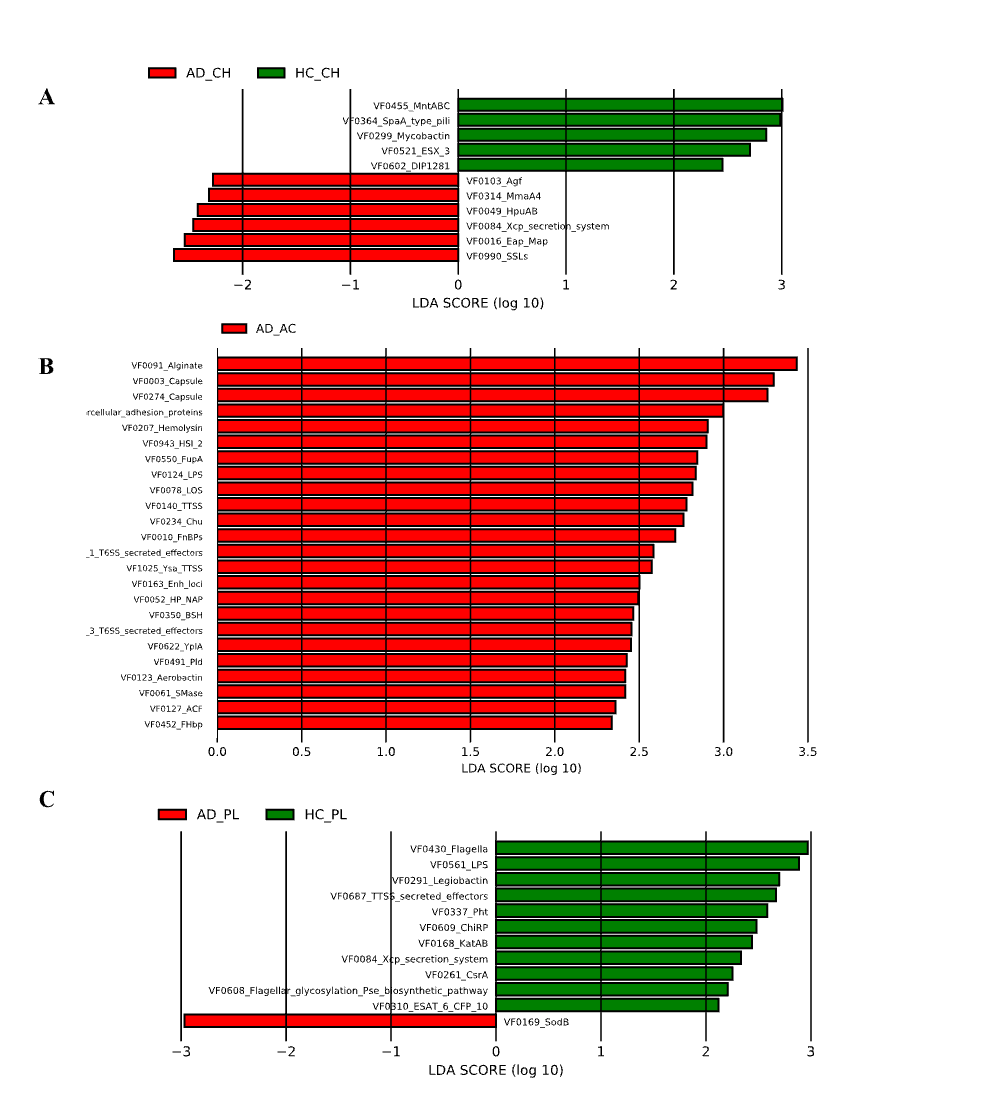


Figure S9. Virulence factor enrichment analysis in samples from (A) the cheek, (B) the antecubital fossa, and (C) the popliteal fossa of atopic dermatitis (AD) patients compared to healthy controls. The bar chart represents the enrichment status; a higher bar indicates a greater difference in the enriched features between the compared groups. AD, atopic dermatitis; HC, healthy control; CH, cheek samples; AC, antecubital samples; PL, popliteal samples.


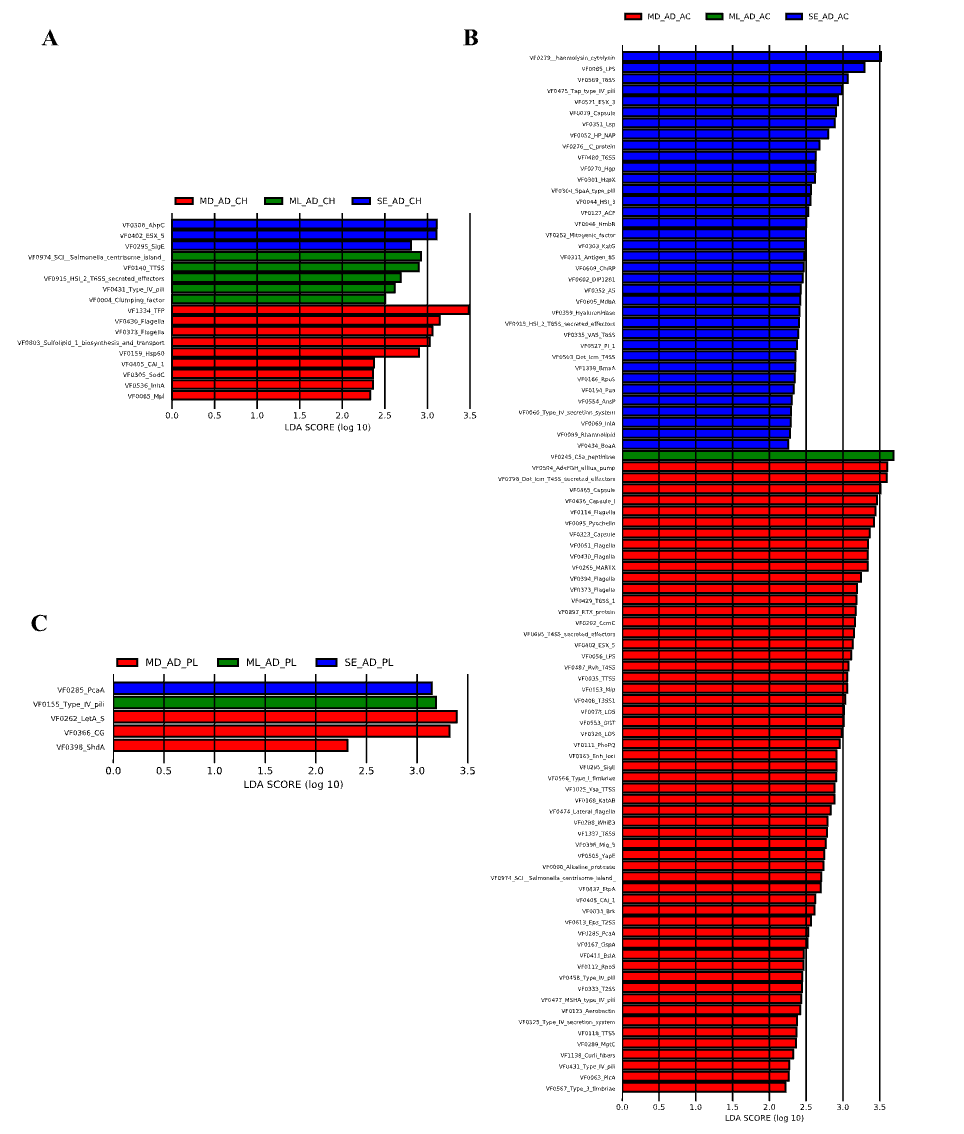


Figure S10. Virulence factor enrichment analysis comparing different disease severities in samples from (A) the cheek, (B) the antecubital fossa, and (C) the popliteal fossa of patients with atopic dermatitis (AD). The bar chart represents the enrichment status; a higher bar indicates a greater difference in the enriched features between the compared groups. AD, atopic dermatitis; HC, healthy control; ML, mild; MD, moderate; SE, severe; CH, cheek samples; AC, antecubital samples; PL, popliteal samples.


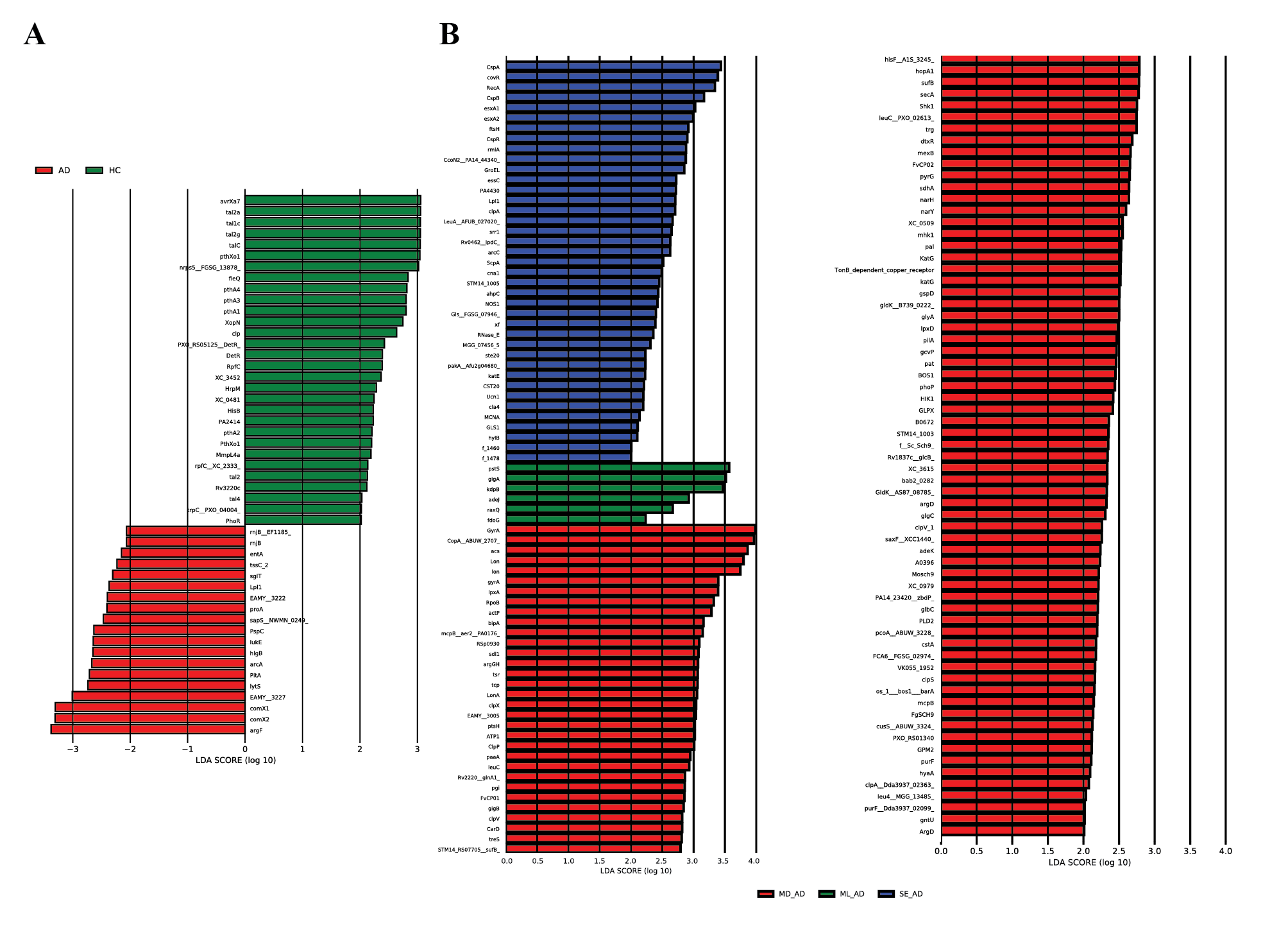


Figure S11. Enrichment of pathogen host interaction genes in samples from patients with atopic dermatitis (AD). (A) Enrichment of microbial pathogen host interaction genes in overall samples from patients with AD compared with healthy controls. Red bars indicate genes significantly enriched in the AD group, whereas green bars indicate those enriched in healthy samples. Nineteen genes were significantly enriched in the AD group. (B) Enrichment of microbial pathogen host interaction genes in overall samples across mild, moderate and severe AD groups. Different colors represent genes enriched in each severity group. The figure shows that the moderate AD group exhibited the greatest number of enriched genes overall, with 99 in total. AD, atopic dermatitis; HC, healthy control; ML, mild; MD, moderate; SE, severe.


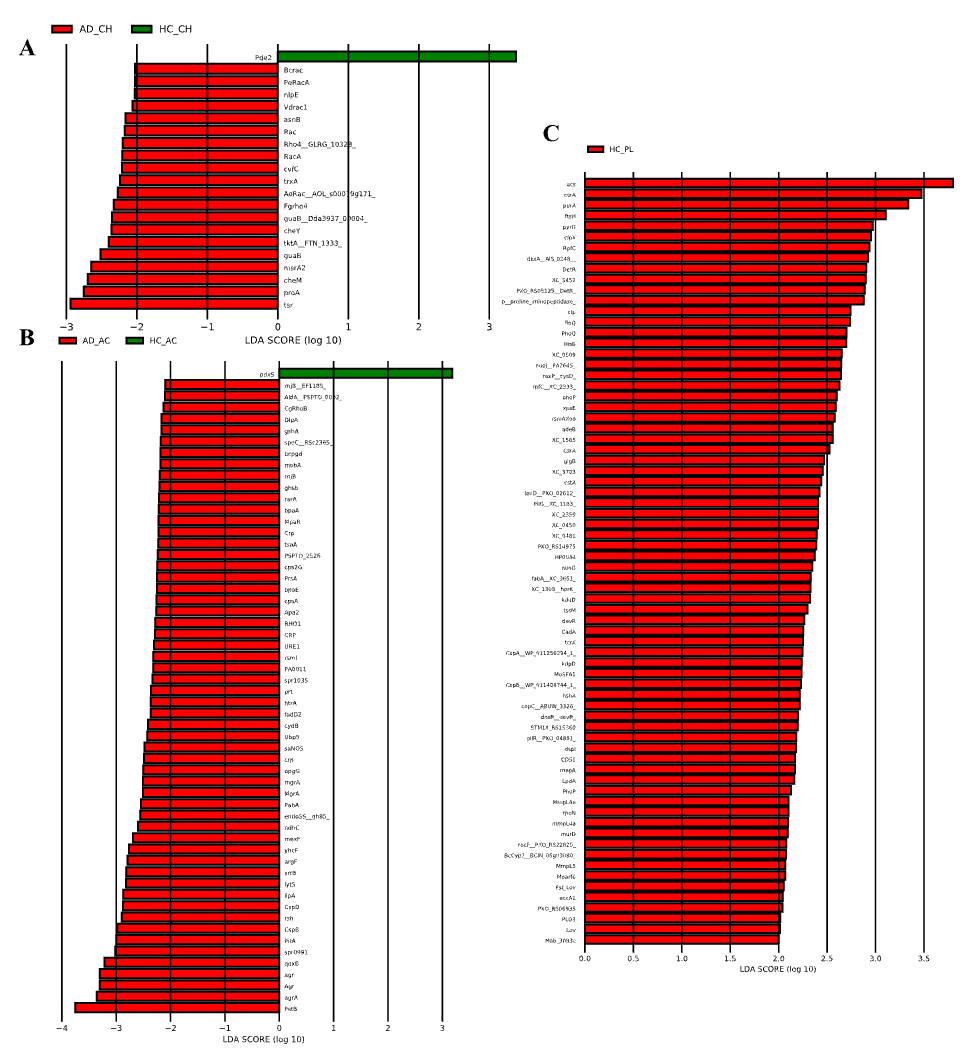


Figure S12. Pathogen-host interaction enrichment analysis in samples from (A) the cheek, (B) the antecubital fossa, and (C) the popliteal fossa of atopic dermatitis (AD) patients compared to healthy controls. The bar chart represents the enrichment status; a higher bar indicates a greater difference in the enriched features between the compared groups. AD, atopic dermatitis; HC, healthy control; CH, cheek samples; AC, antecubital samples; PL, popliteal samples.


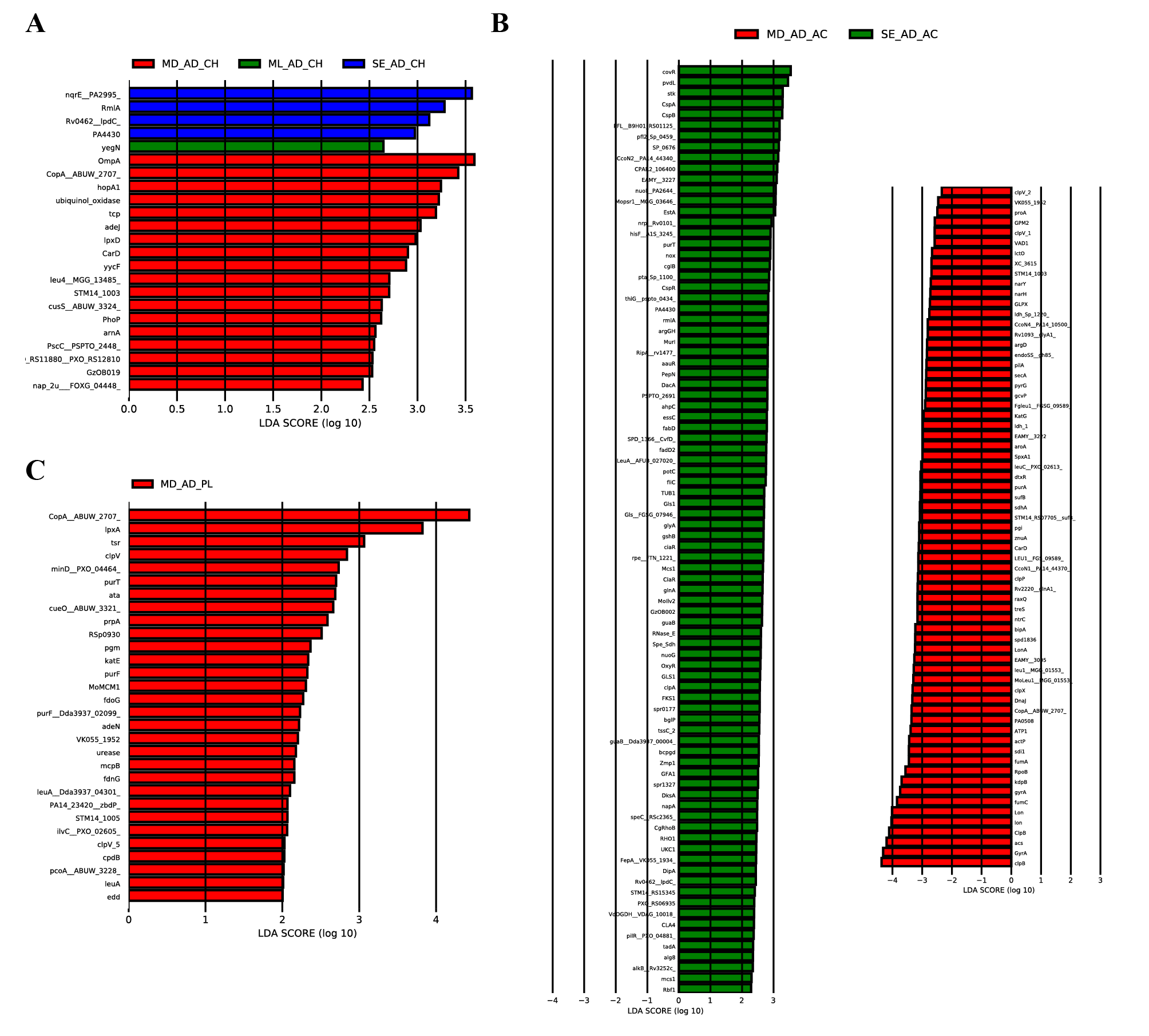


Figure S13. Pathogen-host interaction enrichment analysis comparing different disease severities in samples from (A) the cheek, (B) the antecubital fossa, and (C) the popliteal fossa of patients with atopic dermatitis (AD). The bar chart represents the enrichment status; a higher bar indicates a greater difference in the enriched features between the compared groups. AD, atopic dermatitis; HC, healthy control; ML, mild; MD, moderate; SE, severe; CH, cheek samples; AC, antecubital samples; PL, popliteal samples.
